# The performance of phenomic selection depends on the genetic architecture of the target trait

**DOI:** 10.1007/s00122-021-03997-7

**Published:** 2021-11-22

**Authors:** Xintian Zhu, Hans Peter Maurer, Mario Jenz, Volker Hahn, Arno Ruckelshausen, Willmar L. Leiser, Tobias Würschum

**Affiliations:** 1 Institute of Plant Breeding, Seed Science and Population Genetics, University of Hohenheim, 70593 Stuttgart, Germany; 2 State Plant Breeding Institute, University of Hohenheim, 70593 Stuttgart, Germany; 3 Hochschule Osnabrück, Sedanstr. 26, 49076 Osnabrück, Germany

## Abstract

**Key message:**

The phenomic predictive ability depends on the genetic architecture of the target trait, being high for complex traits and low for traits with major QTL.

**Abstract:**

Genomic selection is a powerful tool to assist breeding of complex traits, but a limitation is the costs required for genotyping. Recently, phenomic selection has been suggested, which uses spectral data instead of molecular markers as predictors. It was shown to be competitive with genomic prediction, as it achieved predictive abilities as high or even higher than its genomic counterpart. The objective of this study was to evaluate the performance of phenomic prediction for triticale and the dependency of the predictive ability on the genetic architecture of the target trait. We found that for traits with a complex genetic architecture, like grain yield, phenomic prediction with NIRS data as predictors achieved high predictive abilities and performed better than genomic prediction. By contrast, for mono- or oligogenic traits, for example, yellow rust, marker-based approaches achieved high predictive abilities, while those of phenomic prediction were very low. Compared with molecular markers, the predictive ability obtained using NIRS data was more robust to varying degrees of genetic relatedness between the training and prediction set. Moreover, for grain yield, smaller training sets were required to achieve a similar predictive ability for phenomic prediction than for genomic prediction. In addition, our results illustrate the potential of using field-based spectral data for phenomic prediction. Overall, our result confirmed phenomic prediction as an efficient approach to improve the selection gain for complex traits in plant breeding.

**Supplementary Information:**

The online version contains supplementary material available at 10.1007/s00122-021-03997-7.

## Introduction

Selection approaches in plant breeding have seen a rapid development with the decreasing costs of molecular marker data (Varshney et al. 2020). Identification of functional markers related to quantitative trait loci (QTL) provides the opportunity to utilize marker-assisted selection in breeding, but requires a sufficiently high proportion of genotypic variance explained by the selected markers (Würschum 2012). Consequently, marker-assisted selection is a powerful and valuable approach for traits with major QTL. However, for highly complex inherited traits that are controlled by numerous loci with small effects, these QTL cannot be identified and thus utilized for selection. Genomic selection is an alternative approach to assist selection for such complex traits. It jointly considers all markers in the entire genome and is thereby able to also capture the effects of small-effect loci (Meuwissen et al. 2001; Heffner et al. 2009). Over the last twenty years, genomic selection has proven to be a promising approach for the prediction of complex traits, as for example demonstrated for grain yield in various crops (Zhao et al. 2012, 2015; Würschum et al. 2013; Pace et al. 2015; He et al. 2016; Thorwarth et al. 2017; Zhu et al. 2021). Nevertheless, in practical breeding programs, it is often challenging to obtain the required high-throughput genotypic data in each generation for a large number of newly established progeny, mainly due to the associated costs (Crossa et al. 2017).

Recently, phenomic selection has been suggested as an alternative to genomic selection in plant breeding (Rincent et al. 2018). Rincent et al. (2018) used near-infrared reflectance spectroscopy (NIRS) data as predictors and reported phenomic predictive abilities as high as those obtained with marker data. NIRS data are routinely collected in breeding programs to estimate the content of seed components such as water content, or protein and oil content (Font et al. 2006; Cen et al. 2007). Moreover, high-throughput phenotyping platforms or unmanned aerial vehicles nowadays enable the breeder to collect spectral data in the field at large scale (Montes et al. 2007; White et al. 2012; Busemeyer et al. 2013; Andrade-Sanchez et al. 2014). Thus, the use of spectral data as predictors could drastically increase the efficiency of selection at greatly reduced costs. Phenomic prediction based on NIRS or field-based hyperspectral data was reported for different crops and traits and shown to achieve promising predictive abilities, for example, in soybean (Parmley et al. 2019), maize (Lane et al 2020), wheat (Rincent et al. 2018; Krause et al. 2019), rye (Galán et al. 2020, 2021), and sugarcane (Gonçalves et al. 2021).

Phenomic prediction is still in its infancy compared to genomic prediction and advantages as well as possible limitations for its utilization in breeding need to be further evaluated. The small-grain cereal triticale (× *Triticosecale* Wittmack) is a man-made cross between wheat and rye, and is extensively used for animal feed and bioenergy (Jørgensen et al. 2007; Mergoum et al. 2009). The present study is based on a total of 1216 triticale lines consisting of a diversity panel (*n* = 846) and two doubled haploid populations, DH1 (*n* = 180) and DH2 (*n* = 190). Our objectives were to (1) compare the predictive abilities of phenomic prediction and genomic prediction for yield, yield-related traits and disease resistance traits, (2) investigate the phenomic predictive ability using NIRS data from a single environment to predict trait performance at another environment, (3) evaluate the predictive ability across material groups, and (4) assess the effect of the training set size on the predictive ability. Collectively, our results confirmed phenomic prediction as a valuable tool for the selection of complex traits in plant breeding programs.

## Materials and methods

### Field design and Phenotyping

For the field trials, we used yield plots and observation plots to evaluate yield and yield-related traits or disease resistance traits, respectively. The total of 1280 triticale genotypes were divided into two trials: trial 1 with 800 lines and trial 2 with 500 lines, with 20 common genotypes as checks (Table S1). The yield plots of trial 1 were evaluated in 2014 and 2015 and trial 2 in 2015 at five locations in southern Germany. The size of the yield plots varied among the locations, ranging from 5 to 10.5 m^2^. A partially replicated (p-rep) design was used for both trials, with a replication rate of 1.3 in trial 1 and 1.2 in trial 2. As for the observation plots, both trial 1 and trial 2 were grown in 2015 at three locations. Each plot consisted of three rows of length 1.25 m and spacing at 0.17 m between rows. All genotypes in both trials were evaluated in a randomized incomplete block design with two replications. The observation plots were not treated with fungicides to enable disease scorings.

The five traits grain yield, thousand-kernel weight, plant height, powdery mildew, and yellow rust were used in this study. The yield plots were harvested and grain yield was calculated at a moisture content of 14%. Thousand-kernel weight was evaluated by a MARVIN seed analyzer (GTA Sensorik GmbH). Plant height was evaluated in the yield plots and the observation plots, by measuring the height after flowering from the ground to the tip of the ears, excluding awns. Powdery mildew and yellow leaf rust infestation was scored in the field on a 1 (completely healthy) to 9 (completely infected) scale.

In the end, 1216 genotypes were used for further analysis since these genotypes had genotypic data available. These consisted of 846 lines from the diversity panel (registered cultivars, *n* = 129; advanced breeding lines, *n* = 717; 787 in trial 1 and 79 in trial 2 with 20 common genotypes) and two doubled haploid populations, DH1 (Modus × Agrano, *n* = 180; 1 in trial 1 and 179 in trial 2) and DH2 (Corino × Witon, *n* = 190; 6 in trial 1 and 184 in trial 2).

### Phenotypic data analysis

Previous studies (Neuweiler et al. 2020, 2021; Trini et al. 2021) have reported grain yield, thousand-kernel weight and plant height based on the same field experiment. For the other traits, the Bonferroni–Holm test was used for outlier detection (Bernal-Vasquez et al. 2016). The best linear unbiased estimates (BLUEs) of each genotype for traits across environments (location-year combinations) were estimated by the following model:

1 $$y_{ijklm} = \mu + g_{i} + e_{j} + \left( {ge} \right)_{ij} + \left( {et} \right)_{jk} + r_{jkl} + b_{jklm} + \varepsilon_{ijklm}$$

where $y_{ijklm}$ is the observed phenotypic data for each plot $\mu$ the overall mean, ${g}_{i}$ the effect of the *i*th genotype, ${e}_{j}$ the effect of *j*th environment, ${(ge)}_{ij}$ the genotype-by-environment interaction effect between the *i*th genotype and the *j*th environment, ${(et)}_{jk}$ the environment-by-trial interaction effect between the *j*th environment and the *k*th trial, ${r}_{jkl}$ the *l*th replication within the *j*th environment and *k*th trial, ${b}_{jklm}$ the *m*th block within the *l*th replication, *j*th environment and *k*th trial, and ${\varepsilon }_{ijklm}$ is the residual error. Heterogeneous error variances were assumed for replications, blocks, trials and environments. To estimate the BLUEs, ${g}_{i}$ was treated as a fixed term.

For the estimation of BLUEs at each single environment, model (1) was modified by removing the environment term:

2 $$y_{ijkl} = \mu + g_{i} + t_{j} + r_{jk} + b_{jkl} + \varepsilon_{ijkl}$$

Some environments only had one trial, e.g., all five locations in 2014, and thus a simpler statistical model without trial factor was used:

3 $$y_{ijk} = \mu + g_{i} + r_{j} + b_{jk} + \varepsilon_{ijk}$$

Heritability was estimated by the regression approach described in Schmidt et al. (2019), as ${\text{BLUP}}_{i } = H_{reg}^{2} \left( {{\text{BLUE}}_{i} - \hat{\mu }_{r} } \right) + \varepsilon_{i}$, where the ${\text{BLUP}}_{i }$ is the best linear unbiased predictor of the *i*th genotype, estimated by a model treating $g_{i}$ as a random term, ${\text{BLUE}}_{i}$ the adjusted mean of the *i*th genotype, ${\widehat{\mu }}_{r}$ the estimate of the intercept, and ${\varepsilon }_{i}$ is the vector of errors of ${\mathrm{BLUP}}_{i}$. For the diversity panel, DH1, and DH2, the BLUPs and BLUEs of genotypes were used to estimate the heritability. All mixed model calculations were performed with ASReml3 (Butler et al. 2009).

### NIRS data and hyperspectral data

**Fig. 1:**
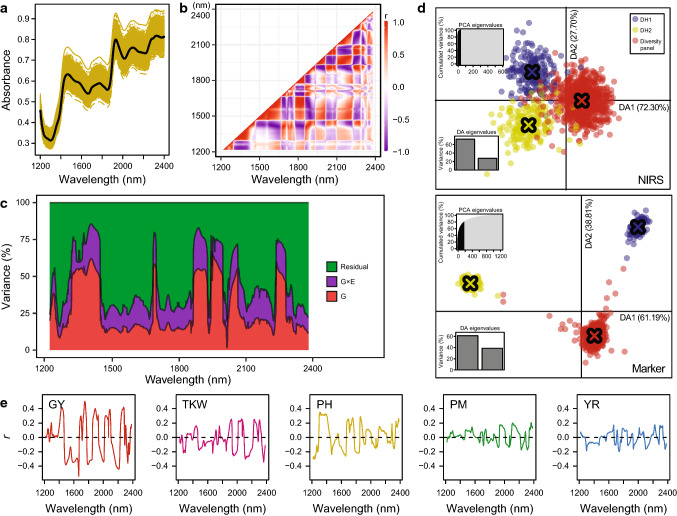
Characterization of the NIRS data. **a** Raw NIRS profiles of triticale grain samples from HOH in 2014. The black line shows the average and the yellow lines are individual genotypes that illustrate the variation. **b** Correlations among all 1165 wavelengths. **c** Proportion of genotypic, genotype-by-environment interaction and residual variance of each wavelength along the NIR spectrum. **d** Discriminant analysis of principal components (DAPC) scatter plot of 1216 triticale genotypes based on NIRS data or molecular markers. Three groups are shown: the diversity panel (*n* = 846), and the doubled haploid populations DH1 (*n* = 180) and DH2 (*n* = 190). The top left inset shows the variance explained by retained principal components and the inset bottom left graph shows the variance explained by the discriminant functions. The crosses refer to the center of each group. **e** Correlation (*r*) between the traits grain yield (GY), thousand-kernel weight (TKW), plant height (PH), powdery mildew (PM) and yellow rust (YR) BLUEs and NIRS BLUEs across environments shown along the entire spectrum

Near-infrared spectroscopy (NIRS) data were obtained from five locations in both 2014 and 2015, except MSB and FCA for trial 1 in 2015 (Table S1). After harvesting, the grains were dried to a uniform moisture content of approximately 14% and then analyzed using a stationary NIRS instrument (Model PSS-X-212, Polytec GmbH, Germany). Intensity of reflectance was measured for each wavelength in the spectral range from 1200 to 2400 nm with a step size of 1 nm. Note that in contrast to marker data where we have two genotypic classes (or three if heterozygotes are present) at each marker, for NIRS data, we have a continuous distribution of the reflectance values at each wavelength and thus many different states (Fig. 1a). Three NIRS measurements were taken per sample. We then performed a principal component analysis to identify and remove outlier spectra. Then, the means of the measurements per sample were calculated and used for further analysis. The first derivative was computed from the NIRS data using a Savitzky–Golay algorithm with the R package ‘prospectr’ function ‘savitzkyGolay’ (Stevens and Ramirez-Lopez 2020) and the window size was set as 37, resulting in 1165 wavelengths used for subsequent analyses (Fig. S1). The BLUEs of each wavelength were calculated across environments and within each environment based on model (1) or model (2–3).

Hyperspectral imaging data were collected at some locations in 2014 and 2015 at one to three time points (Table S1). In 2014, hyperspectral data consisted of 250 wavelengths ranging from 976.1 to 1689.4 nm with a width of around 2.9 nm (HELIOS Core NIR, EVK DI KERSCHHAGGL GmbH, Austria) obtained by the BreedVision platform (Busemeyer et al. 2013). In 2015, hyperspectral data consisted of 248 wavelengths ranging from 930.0 to 1700.0 nm with a width of around 3.1 nm (HELIOS NIR G2-320, EVK DI KERSCHHAGGL GmbH, Austria). Raw data were first filtered to pixels of plants using a spectral angle mapping approach (Kruse et al. 1993) and then processed with the same approach as the NIRS data. BLUEs of each wavelength were only calculated at a single environment at each time point using model (3), yielding a total of 214 wavelengths in 2014 and 212 wavelengths in 2015 that were used as predictors in the further analysis.

### Genotypic data analysis

All individuals were genotyped by a genotyping-by-sequencing approach (DArTseq) at Diversity Arrays Technology Pty. Ltd. Quality checks were performed separately in the diversity panel and the DH1 and DH2 populations. For the diversity panel, markers with a missing rate higher than 0.2 or a minor allele frequency (MAF) lower than 0.03 were discarded. Imputation was performed with LinkImpute (Money et al. 2015). After imputation, markers with MAF lower than 0.05 were removed. Finally, 38,583 markers were obtained (31,045 dominant silico-DArTs and 7,538 SNPs). For the two biparental families, the markers remaining for the diversity panel were chosen, and imputation was performed separately in each family.

Population structure analysis using NIRS data and molecular marker data was done with discriminant analysis of principal components in the R package ‘adegenet’ with the ‘dapc’ function (Jombart et al. 2008, 2010).

### Detection of major QTL

Genome-wide association analysis was performed separately within the diversity panel, DH1, and DH2. Marker data were filtered for MAF of 0.05 for the diversity panel and 0.2 for the DH1 and DH2 populations. The mixed linear model (MLM) approach including a kinship matrix was used for analysis using Tassel 5 (Yu et al. 2006; Bradbury et al. 2007). The exploratory significance threshold of −log_10_(*p-value*) of 4 was chosen and the explained phenotypic variance (*R*^2^) was calculated by fitting all significant markers in the order of their strength together in a joint model. Markers with *R*^2^ above eight percent were selected as major QTL and used in further analysis.

### Models for prediction

The widely used approach ridge regression best linear unbiased prediction (RR-BLUP) was used as implemented in the R package ‘rrBLUP’ (Endelman et al. 2011). Regarding the different predictor categories used in this study, we compared models with (1) only NIRS data as random effect; (2) only genome-wide molecular marker data as random effect; (3) only major QTL; (4) major QTL as fixed effect plus NIRS data as random effect; (5) major QTL as fixed effect and remaining markers as random effect; (6) only hyperspectral imaging data as random effect. Models (1–2) and (4–6) were implemented with the R package ‘rrBLUP,’ and model (3) with the regression linear model using the R function ‘lm.’

The performance of models was evaluated by the predictive ability that is the Pearson’s correlation coefficient between the BLUEs and the estimated performance values. We used fivefold cross-validation with 1000 runs.

## Results

This study was based on 1216 triticale lines comprising of a diversity panel and two doubled haploid populations (DH1 and DH2) with 846, 180, and 190 lines, respectively. All lines were evaluated for grain yield, thousand-kernel weight, plant height, powdery mildew, and yellow rust in multi-environment field trials (Table S1, S2).

The raw NIRS data were obtained from harvested and dried seeds and showed substantial variation (Fig. 1a). The heatmap of the correlations among the wavelengths showed several wavelength clusters (Fig. 1b). Estimation of the variance components along the entire spectrum revealed that the genotypic variance accounted for over 50% in some wavelengths regions and generally had a similar or higher proportion than the genotype-by-environment interaction variance (Fig. 1c). Discriminant analysis of principle components based on the adjusted NIRS BLUEs or molecular marker data showed a high explained variance of the first two discriminant functions. The three groups were separated more clearly based on the marker data (Fig. 1d). The correlations between across-environment BLUEs of the traits and the NIRS data revealed the strongest and on average highest correlations for grain yield, lower ones for thousand-kernel weight and plant height, and the lowest correlations for powdery mildew and yellow rust (Fig. 1e).

**Fig. 2:**
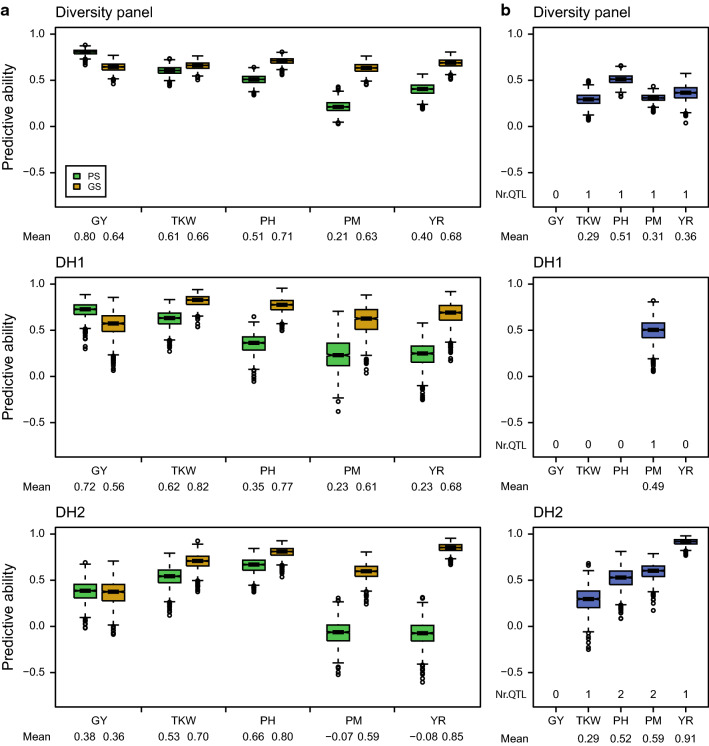
Dependency of phenomic prediction on the genetic architecture of the target trait. **a** Predictive ability of phenomic prediction (PS) and genomic prediction (GS) for grain yield (GY), thousand-kernel weight (TKW), plant height (PH), powdery mildew (PM) and yellow rust (YR) shown for the diversity panel and the two doubled haploid populations (DH1, DH2). Results were obtained from 1000 runs of fivefold cross-validation. **b** Predictive ability of major QTL explaining more than eight percent of the phenotypic variance

We used the NIRS data as predictors for phenomic prediction and compared the predictive abilities of the five traits in each group with those obtained by genomic prediction (Fig. 2a). For grain yield, the average predictive ability was higher for phenomic prediction than for genomic prediction in all three groups: 0.80 compared to 0.64 in the diversity panel, 0.72 compared to 0.56 in DH1, and 0.38 compared to 0.36 in DH2. For thousand-kernel weight and plant height, the phenomic predictive abilities were generally slightly lower than the genomic predictive abilities. For the two disease resistance traits powdery mildew and yellow rust, the genomic predictive abilities ranged between 0.59 and 0.85 in each of the groups. By contrast, the phenomic predictive abilities were much lower, often around 0.2 or even close to zero in DH2.

This prompted us to investigate the genetic architecture of these traits. QTL explaining more than 8% of the phenotypic variance were declared as medium- to major-effect QTL and used to assess their predictive ability (Fig. 2b; Table S3). Note that the lowest predictive ability is then 0.28 as determined by the square root of the 8% explained variance of QTL selected as threshold. No QTL were identified for grain yield in all three groups, confirming it to be a complex trait controlled by numerous small-effect loci. As for the other four traits, we identified QTL in at least two of the groups with predictive abilities ranging from 0.29 to 0.91. The latter was observed for a yellow rust QTL in DH2 that explained 85.5% of phenotypic variance and consequently yielded a predictive ability of 0.91. In this case, this single marker achieved a comparable predictive ability as genomic prediction, whereas in this situation, the phenomic predictive ability was close to zero (Fig. 2a).

In addition, we used these QTL as fixed effects in the prediction model together with either NIRS data or marker data as random effect to construct two weighted models and compared their performance with the fully random models. The model incorporating QTL and marker always achieved the highest predictive ability among all models (Fig. S2). Apart from exceptions like yellow rust in DH2, the inclusion of QTL improved the phenomic and genomic predictive abilities only slightly.

**Fig. 3:**
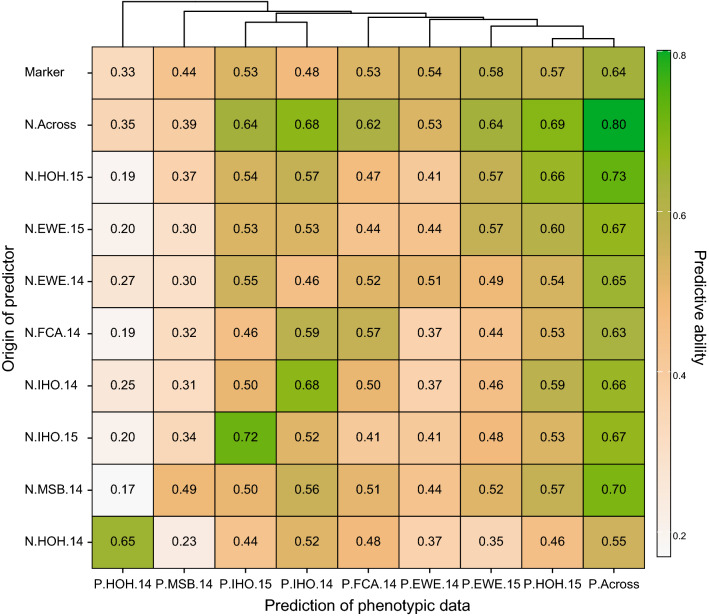
Effect of the environmental origin of NIRS data on the phenomic predictive ability. NIRS BLUEs from each of the eight environments and across these environments were used as predictors. Phenotypic data were the grain yield BLUEs from each of the environments and across environments that were predicted by cross-validation using NIRS BLUEs from the same or another environment. For comparison, the genomic predictive ability is shown. Means from 1000 runs of fivefold cross-validation are shown. The clusterplot at the top shows the correlations among grain yield BLUEs from each of the environments and across environments

These findings substantiated the potential of phenomic prediction especially for complex traits like grain yield. We therefore investigated the predictive ability of NIRS data for grain yield in the diversity panel, when the NIRS and the trait values are derived from different environments. As a reference, the phenomic predictive ability obtained by cross-validation within the respective environment can be used, and in addition, the results from genomic prediction are shown for comparison (Fig. 3). For the prediction of grain yield performance across environments, NIRS data across environments as predictors achieved the highest predictive ability and most NIRS data from a single environment performed better than or comparable with marker data, except for the NIRS data from HOH in 2014. As for the prediction of grain yield within single environments, our result showed that NIRS data from the same environment as the trait data or NIRS data across environments resulted in higher or comparable predictive abilities as molecular marker data. Grain yield at the locations HOH and MSB in 2014 had a much lower correlation with the other environments and the predictive abilities for grain yield at these two environments with NIRS data from the other environments were also lower than those obtained for the same scenario at other environments (Fig. 3, S3). For these two environments, the genomic predictive abilities were also lower than for the other environments.

**Fig. 4:**
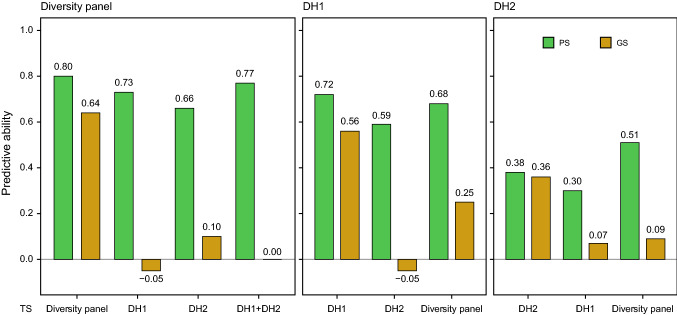
Phenomic prediction for grain yield among groups of breeding material. Phenomic (PS) and genomic prediction (GS) of the diversity panel using one or both doubled haploid populations (DH1, DH2) as training set (TS) or of either of the two doubled haploid populations using the other doubled haploid population or the diversity panel as training set. As reference, the cross-validated predictive ability within the respective group is shown

The genetic relatedness between the training set and the prediction set is a key factor for the success of genomic prediction in plant breeding (Brauner et al. 2020; Zhu et al. 2021). We therefore further investigated the predictive abilities of phenomic prediction and genomic prediction for grain yield among the three groups, i.e., among the diversity panel and the two DH populations (Fig. 4). The average cross-validated predictive ability within each group serves as a reference. For prediction in the diversity panel, the within-group predictive ability was 0.80, and only slightly lower predictive abilities were obtained when the DH populations were used as training set, with 0.73 for DH1 and 0.66 for DH2. When both DH populations were jointly used as training set, the predictive ability in the diversity panel was 0.77. By contrast, the genomic predictive ability was 0.64 within the diversity panel, but −0.05 and 0.10 when DH1 or DH2, respectively, were used as training set and zero for a training set comprising both populations. For the two DH populations the picture was similar, with phenomic predictive abilities comparable to the within-group reference value when the other DH population or the diversity panel were used as training set. Again, the genomic predictive abilities were much lower, mostly below 0.10.

**Fig. 5:**
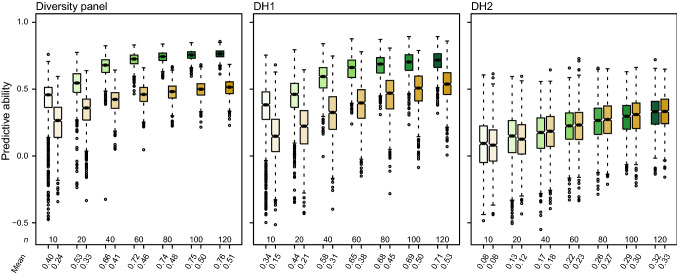
Predictive ability and training set size. The results for phenomic prediction (green) and genomic prediction (brown) are shown for grain yield and were obtained from 1000 runs of fivefold cross-validation

The optimization of the training set size is essential for plant breeding as it affects the predictive ability as well as the costs. Hence, we investigated the effect of the training set size on the phenomic and genomic predictive ability for grain yield in each of the three groups (Fig. 5). We observed an increase in the predictive abilities of phenomic and genomic prediction with increasing size of the training set in all three groups. In the diversity panel, both approaches showed a similar trend with the predictive ability increasing up to around 60 individuals and then starting to plateau. In DH1, the phenomic predictive ability also started to plateau after a training set size of 60 individuals, whereas the genomic predictive ability showed a more continuous increase. Within the diversity panel and DH1, phenomic prediction with a training set size of only 20 to 40 could obtain a predictive ability as high as that from genomic prediction with 120 individuals in the training set. For DH2, by contrast, both approaches were on the same level and the predictive ability increased continuously.

**Fig. 6:**
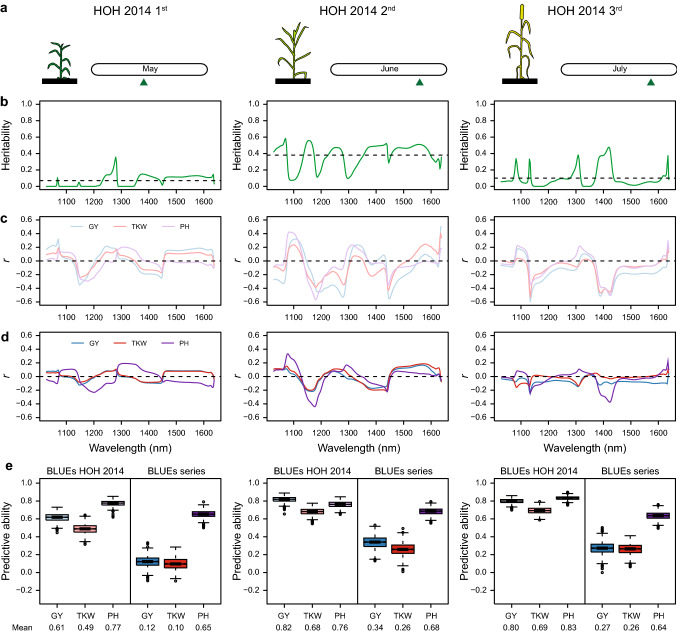
Phenomic prediction with field-based hyperspectral imaging data. **a** Date of hyperspectral data collection at HOH in 2014 for three measurement dates in the field (first, second, third) (green triangles). **b** Heritability along the hyperspectral profile shown for these three measurement dates. **c** Correlation (*r*) between hyperspectral data of each measurement date and grain yield BLUEs of the same environment HOH 2014. **d** Correlation between hyperspectral data of each measurement date and grain yield BLUEs from the series across all environments. **e** Predictive ability of hyperspectral data from these three measurement dates for grain yield (GY), thousand-kernel weight (TKW) and plant height (PH) shown for the trait BLUEs within this environment (BLUEs HOH 2014, left box) and the BLUEs from the series across all environments (BLUEs series, right box). The results were obtained from 1000 runs of fivefold cross-validation

Last, we evaluated the potential of field-based hyperspectral imaging data for phenomic prediction. For this, we replaced the NIRS predictors in phenomic prediction by hyperspectral imaging data collected at different developmental stages of the plants, and in the diversity panel assessed the predictive ability for grain yield, thousand-kernel weight, and plant height within this environment or across environments (Fig. 6, Fig. S4). For the location HOH in 2014, there were three time points of measurement that approximately correspond to the developmental stages of the flag leaf becoming visible (BBCH 37), ear emergence (BBCH 51) and beginning of milk development (BBCH 70). The heritability of the hyperspectral data varied along the spectrum as well as among the three time points. Likewise, the correlations between hyperspectral data and trait values varied. The predictive abilities for grain yield and thousand-kernel weight of within-environment trait values were much higher than the prediction of across-environment trait values. When predicting grain yield within HOH in 2014, the phenomic predictive abilities ranged from 0.61 to 0.82 for the three time points. By contrast, the same hyperspectral data only achieved predictive abilities of 0.12 to 0.34 for grain yield across environments. As for the prediction of plant height, the predictive abilities were all high even for the across-environment performance, ranging from 0.64 to 0.83. We also used hyperspectral data from other environments to predict the three traits within that environment or across environments (Fig. S4). The predictive ability for grain yield varied from 0.10 to 0.64 for the within-environment prediction and from 0.16 to 0.56 for the prediction of the across-environment performance. In 2015, we changed the phenotyping platform, and at three locations, the hyperspectral data achieved similarly high predictive abilities ranging from 0.49 to 0.64 for grain yield within as well as across environments.

## Discussion

Phenomic prediction has been suggested as an alternative to genomic prediction and the predictive abilities reported for important agronomic traits as well as the lower costs compared to genomic selection illustrate its potential for plant breeding (Rincent et al. 2018). The aim of this study was to evaluate the use of phenomic prediction in triticale, but mainly to further explore the utilization of this approach for plant breeding.

### Phenomic prediction using NIRS data as predictors is promising for complex traits

Our result showed that the predictive ability of phenomic prediction based on NIRS data for grain yield in a diversity panel and two DH populations of triticale was similar or superior to that of genomic prediction (Fig. 2a). This corroborates the results of previous studies that illustrated the potential of phenomic prediction using spectral data to predict complex traits in different crops (Rincent et al. 2018; Krause et al. 2019; Galán et al. 2021).

Interestingly, we observed comparably low predictive abilities for the two disease resistance traits, particularly for yellow rust in one of the DH populations. QTL mapping identified a QTL explaining 85% of the phenotypic variance, illustrating that in this population the trait is probably oligogenic but mainly controlled by a single major QTL. This QTL can be captured in marker-assisted selection, but also by genomic prediction. For the latter, each marker can only capture a small part of the variance of this QTL, but if enough markers are available in the QTL region, they can jointly capture the full variance of such a major QTL and allow accurate predictions. Phenomic prediction is expected to exploit endophenotypes by associating them with the target traits and similar to genomic prediction may profit from overall similarities and relatedness. Thus, if two lines differ at a single locus, this cannot be differentiated by phenomic prediction. Phenomic prediction could only capture such a major QTL if it was related to a specific signal in the NIR spectrum. In conclusion, an essential determinant for the predictive ability of phenomic prediction appears to be the genetic architecture of the target trait. Phenomic prediction is an effective and promising tool for the improvement of complex traits, but is not suited for mono- or oligogenic traits. The latter can, however, also easily be selected for phenotypically or by marker-assisted selection after identification of the major QTL. From a breeding perspective, the potential to predict complex traits like grain yield in a cheap and high-throughput manner is much more valuable.

We also evaluated a model combining QTL and NIRS data, which often achieved similar or better results than the QTL or NIRS data alone. Thus, this combined approach appears suitable for the prediction of traits controlled by one or a few medium- to large-effect QTL and many additional small-effect QTL.

### Using NIRS data from single environments in plant breeding

Unlike molecular markers, NIRS data are affected by the environment and the NIRS profile of a single genotype is not constant from one environment to another. Nevertheless, NIRS data from a single environment can be used for prediction at another environment (Fig. 3). Depending on the similarity of the environments, this yielded predictive abilities that were often almost as high as those obtained using the NIRS data from the same environment as the trait data to be predicted. Moreover, with the exception of the NIRS data from HOH in 2014, the NIRS data from the other seven environments achieved predictive abilities for grain yield across environments comparable to or even higher than genomic prediction. This corroborates previous results and illustrates the suitability of NIRS data from a single environment for phenomic prediction (Rincent et al. 2018). The findings that the predictive ability is the highest, the more similar the environments are, and also higher than the predictive ability achieved with marker data suggests that the NIR spectra also capture non-additive genetic effects which can be exploited by phenomic prediction.

### Negligible effect of genetic relatedness on phenomic prediction based on NIRS data

The accuracy of genomic prediction is known to depend on the genetic relatedness of the individuals in the training and prediction sets (Lehermeier et al. 2014; Würschum et al. 2017; Brauner et al. 2020; Zhu et al. 2021). Predictions among the three groups revealed substantial differences between the phenomic and the genomic approach. Genomic prediction failed when predicting from one group to another as only very low predictive abilities were obtained. By contrast, phenomic prediction achieved among-group predictive abilities that were almost as high as the cross-validated within-group predictive ability that can serve as a reference.

In plant breeding programs, the aim is usually to predict the performance of newly established progeny from the crosses that are initiated each year. For genomic prediction, this is best achieved using full-sibs, i.e., some individuals from a biparental family to predict the remaining ones. However, this can cause a delay and problems in the logistics of a breeding program and in addition requires the phenotyping of a subset of lines from each cross. Our results illustrate the advantage of phenomic prediction, as also other biparental families can be used as training set and can achieve similar predictive abilities. To buffer the effect of a single family, it is recommended to combine several families in composite training sets. In addition, a diversity panel, which in a breeding context would be the entire breeding pool, can be used as training set. Thus, if no large enough biparental families are available to serve as training set, several smaller ones can be combined or diverse breeding material be used as training set for phenomic prediction.

Conversely, also biparental families can make up a training set that warrants high phenomic predictive abilities in a diversity panel. This may not occur often, but may be the case when only a small breeding program is available, with few founder lines and some biparental families. In such a case, the latter can be used as training set to predict diverse material in order to increase the genetic diversity of the breeding pool. Another scenario where this may be of relevance is, when diverse material is obtained from elsewhere with just few seeds, which are then propagated and thus grown in the field next to the yield trials of advanced lines from segregating families. Taken together, phenomic prediction is more robust than genomic prediction when predicting among different families or groups of material.

### Optimum design of the training set

Generally, the accuracy of genomic prediction is also largely determined by the size of the training set (Habier et al. 2007; Zhao et al. 2012; Thorwarth et al. 2017; Herter et al. 2019; Zhu et al. 2021). We therefore compared the predictive ability of phenomic and genomic prediction with various training set sizes (Fig. 5). In the diversity panel and in one of the DH populations, phenomic prediction achieved higher predictive abilities than genomic prediction for a given training set size. Consequently, for a similar predictive ability, phenomic prediction requires a much smaller training set. The reason could be that the spectral data have many more possible states per wavelength compared to the two homozygous genotypic classes at a locus, and thus, training sets with smaller size allow a better training of the effects of each predictor in the prediction model for phenomic prediction than for genomic prediction. In conclusion, the possibility to reduce the training set size is another advantage of phenomic prediction that allows to save resources or to increase selection gain by a higher predictive ability.

### Using field-based spectral data for phenomic prediction

In addition to the spectral data from the NIRS measurements of seeds, we also evaluated the potential of field-based hyperspectral imaging data for phenomic prediction. For grain yield and thousand-kernel weight, we found that the field-based hyperspectral imaging data from different time points of a single environment yielded variable predictive abilities (Fig. 6, S4). This is in line with the reported various relationship matrices derived from individual hyperspectral time points (Krause et al. 2019). In our study, the assessment at a later stage tended to provide a higher predictive ability, but the choice of the optimal developmental stage and environmental conditions warrants further research.

Compared with the results from 2014, in 2015, the predictive abilities of the three environments were higher and more stable across them, which may be due to the improvement of the hyperspectral imaging system on the BreedVision platform (Busemeyer et al. 2013). This may indicate that also technical improvements can increase the predictive ability of phenomic prediction based on field-based spectral data.

Interestingly, the predictive abilities for plant height were comparably high in all environments. This might be due to the image acquisition by the BreedVision platform. The sensor platform is adjusted for the height of the plot to be measured. Nevertheless, the plant architecture varies with height, as for example, the flag leaves are closer to the ears for plants with a shorter stature. Thus, different tissues may be captured by the focal point of the hyperspectral imaging system, which might in part be exploited for prediction of plant height.

In summary, spectral data collected in the field can also be used for phenomic prediction to predict complex traits like grain yield. This opens up new possibilities for breeding, as for example, early generations in the breeding process can be assessed and predicted for grain yield. However, further work is required to optimize this approach. As an alternative to phenotyping platforms, unmanned aerial vehicles are a promising option to collect the spectral data, as they allow the high-throughput required in plant breeding programs (Araus and Cairns 2014; Krause et al. 2020).

## Conclusions

Our results show that phenomic prediction based on NIRS data of seeds or field-based spectral data can achieve high predictive abilities for agronomic traits. There is, however, a dependency of this approach on the genetic architecture of the target trait. For traits controlled by one or a few major QTL, phenomic prediction will generally not be suitable. Such QTL need to be targeted by marker-assisted selection, which can also be combined with phenomic prediction. Collectively, our results confirmed that phenomic prediction is a promising high-throughput and cost-efficient method that can be included as a valuable approach in the breeding toolbox for selection of complex traits.

## Supplementary Information

Below is the link to the electronic supplementary material.

Supplementary file1 (PDF 647 kb)

## Declarations

### Availability of data and material

The data that supported the findings of this study are available upon request through a material transfer agreement. The material of this study can in some cases be made available through a material transfer agreement.
