# Supplementary material for: The performance of phenomic selection depends on the genetic architecture of the target trait
Source: Theor Appl Genet. 2021 Nov 22;135(2):653–65. doi: 10.1007/s00122-021-03997-7 (PMC8866387; doi:10.1007/s00122-021-03997-7)
Supplement: Supplementary file 1 — Supplementary file1 (PDF 647 kb) [file 122_2021_3997_MOESM1_ESM.pdf]

The performance of phenomic selection depends on the genetic architecture of the target trait

Xintian Zhu[1,2], Hans Peter Maurer[2], Mario Jenz[2,3], Volker Hahn[2], Arno Ruckelshausen[3], Willmar L. Leiser[2], Tobias Würschum[1]

[1] Institute of Plant Breeding, Seed Science and Population Genetics, University of Hohenheim, 70593 Stuttgart, Germany

[2] State Plant Breeding Institute, University of Hohenheim, 70593 Stuttgart, Germany

[3] Hochschule Osnabrück, Sedanstr. 26, 49076 Osnabrück, Germany

Supplementary material

Table S1 Overview of the field design and data distribution.

**Field design and environmental condition**							**Phenotype**					**Predictors**	
**Plots**	**Year**	**Location**	**Coordinate**	**Altitude (m)**	**Trial 1**	**Trial 2**	**GY**	**TKW**	**PH**	**PW**	**YR**	**NIRS**	**HSI**
Yield plot	2014	Hohenheim (HOH)	48°42'53"N, 9°11'16"E	400	Y	N	Y/N	Y/N	Y/N	N/N	N/N	Y/N	Y/N (3)
	2014	Ihinger Hof (IHO)	48°44'40"N, 8°55'25"E	480	Y	N	Y/N	Y/N	N/N	N/N	N/N	Y/N	Y/N (2)
	2014	Eckartsweier (EWE)	48°31'17"N, 7°52'13"E	140	Y	N	Y/N	Y/N	N/N	N/N	N/N	Y/N	Y/N (2)
	2014	Moosburg (MSB)	48°26'36"N, 11°54'22"E	420	Y	N	Y/N	Y/N	Y/N	N/N	N/N	Y/N	Y/N (2)
	2014	Franconia (FCA)	49°39'58"N, 9°47'30"E	310	Y	N	Y/N	Y/N	Y/N	N/N	N/N	Y/N	N/N
	2015	Hohenheim (HOH)	48°42'53"N, 9°11'16"E	400	Y	Y	Y/Y	Y/Y	Y/Y	N/N	N/N	Y/Y	Y/Y (1)
	2015	Ihinger Hof (IHO)	48°44'40"N, 8°55'25"E	480	Y	Y	Y/Y	Y/Y	Y/Y	N/N	N/N	Y/Y	Y/N (1)
	2015	Eckartsweier (EWE)	48°31'17"N, 7°52'13"E	140	Y	Y	Y/Y	N/Y	Y/Y	N/N	N/N	Y/Y	N/N
	2015	Moosburg (MSB)	48°26'36"N, 11°54'22"E	420	Y	Y	Y/Y	N/Y	Y/Y	N/N	N/N	N/Y	Y/Y (1)
	2015	Franconia (FCA)	49°49'22"N, 10°6'19"E	270	Y	Y	Y/Y	N/Y	Y/Y	N/N	N/N	N/Y	N/N
Observation plot	2015	Hohenheim (HOH)	48°42'53"N, 9°11'16"E	400	Y	Y	N/N	N/N	Y/Y	Y/Y	Y/Y	N/N	N/N
	2015	Ihinger Hof (IHO)	48°44'40"N, 8°55'25"E	480	Y	Y	N/N	N/N	Y/Y	Y/Y	Y/Y	N/N	N/N
	2015	Oberer Lindenhof (OLI)	48°28′49″N, 9°18′56″E	700	Y	Y	N/N	N/N	Y/Y	Y/Y	Y/Y	N/N	N/N

GY, grain yield; TKW, Thousand-kernel weight; PH, plant height; PM, powdery mildew; YR, yellow rust leaf; NIRS, near infrared spectrum; HSI, hyperspectral imaging.

Y, trial was used; N, trial was not used.

Y/Y, the available data from trial 1 and trial 2; Y/N, the available data only from trial 1; N/Y, the available data only from trial 2; N/N, no available data.

Number within bracket for hyperspectral data shows the times of measurement.

Table S2 Heritability of all five traits in the three groups and three traits (GY, TKW, and PH) in single environments.

	GY	TKW	PH	PM	YR
**Population**					
Diversity panel	0.89	0.89	0.96	0.70	0.78
DH1	0.86	0.97	0.86	0.72	0.67
DH2	0.87	0.93	0.89	0.63	0.59
**Environment (location-year combination)**					
HOH.2014	0.23	0.54	0.81		
IHO.2014	0.70	0.89			
EWE.2014	0.62	0.93			
MSB.2014	0.72	0.89	0.94		
FCA.2014	0.64	0.82	0.88		
HOH.2015	0.77	0.96	0.71		
IHO.2015	0.78	0.89	0.82		
EWE.2015	0.77	0.88	0.84		
MSB.2015	0.68	0.87	0.82		
FCA.2015	0.65	0.88	0.83		

GY, grain yield; TKW, Thousand-kernel weight; PH, plant height; PM, powdery mildew; YR, yellow rust leaf

Table S3 Markers identified as QTL and used as fixed effect in each group.

Population	Trait	Marker	Chr.	Pos. (cM)	R^2 (%)	-log10(*P*)	QTL predictive ability
Diversity panel	TKW	D3607678	5B*	106.08	8.74	4.15	0.29
	PH	D3616655	5R*	1372.3	25.88	46.36	0.51
	PM	D4360946	6A	74.35	9.45	6.18	0.31
	YR	D4344835	2R	55.60	13.66	14.77	0.36
DH1	PM	S8535302	2B	79.45	24.52	4.37	0.49
DH2	TKW	D8536403	5R	1079.5	8.35	4.92	0.29
	PH	D8518305	5A	86.60	13.08	4.22	0.52
		D4219588	6A	92.26	14.46	4.00	
	PM	D3623870	1B	91.93	19.60	5.36	0.59
		D3613768	6R	716.6	18.33	5.29	
	YR	D3615693	6R	930.20	85.50	19.37	0.91

GY, grain yield; TKW, Thousand-kernel weight; PH, plant height; PM, powdery mildew; YR, yellow rust leaf

* unmapped marker put on the genetic map based on the linkage disequilibrium with mapped markers

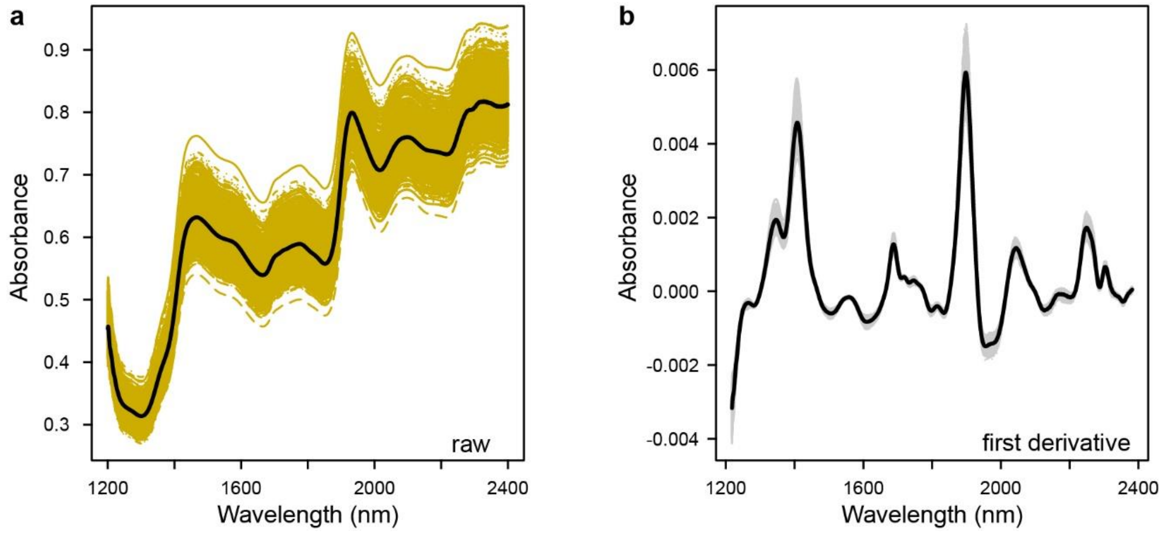

Fig. S1 The NIRS data. **a** Raw NIRS data and **b** normalized first derivative of the NIRS data for the triticale grain samples from HOH in 2014.

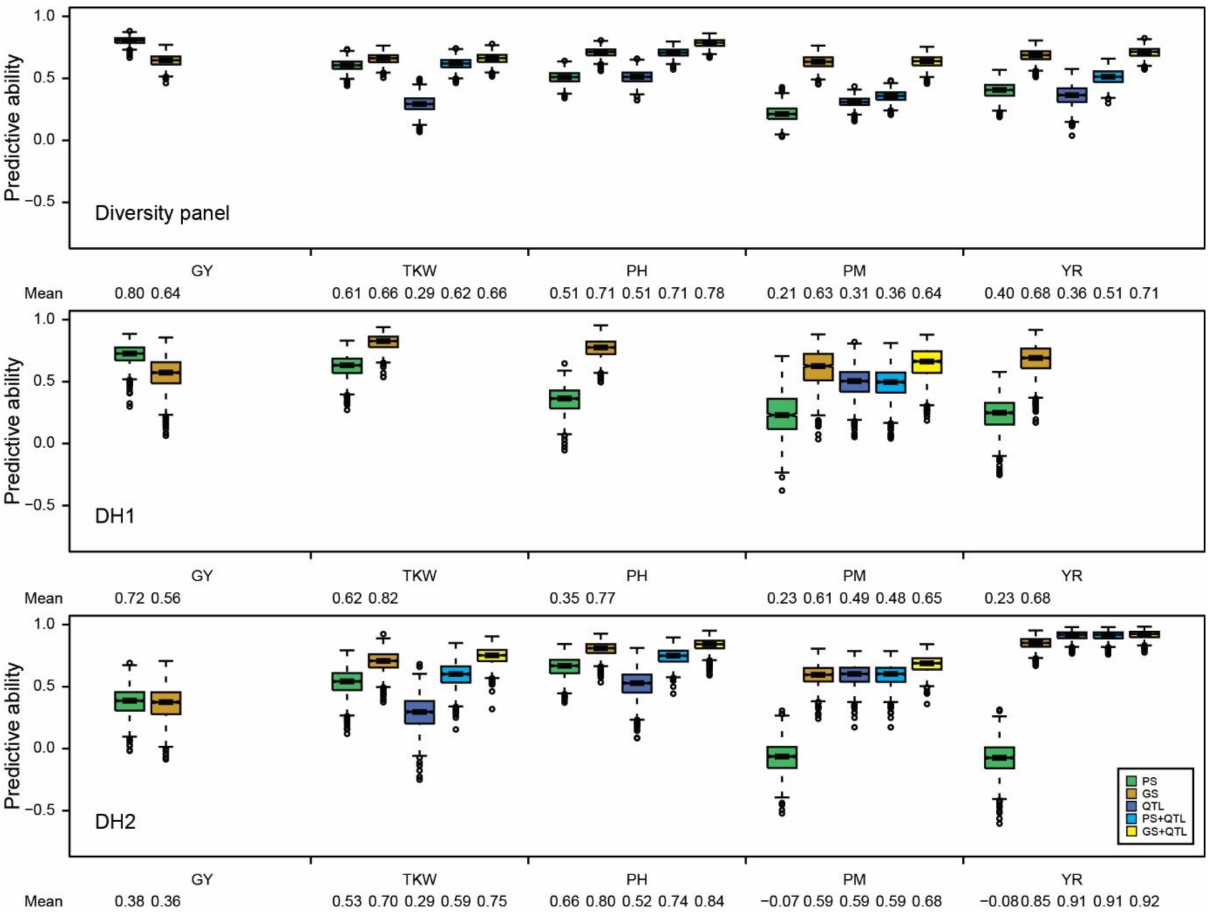

Fig. S2 Boxplot of predictive ability of a model trained with NIRS data (phenomic selection, PS), a model trained with marker data (genomic selection, GS), a model with major QTL (QTL), a model with major QTL as fixed effect and NIRS data as random effect (PS + QTL) and a model with major QTL as fixed effect and remaining markers as random effect (GS + QTL). Result were obtained from 1000 runs of fivefold cross-validation and shown for grain yield (GY), thousand-kernel weight (TKW), plant height (PH), powdery mildew (PM), yellow rust leaf (YR) within the diversity panel and the two doubled haploid populations.

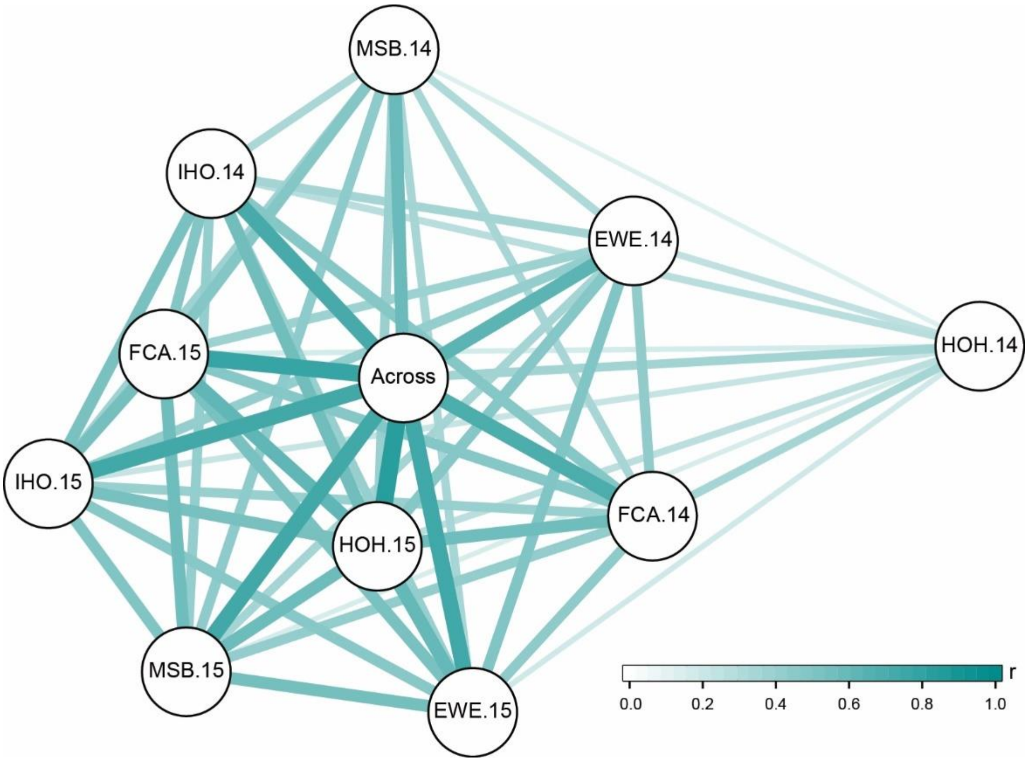

Fig. S3 Network of correlations for grain yield BLUEs from across environments and within each environment.

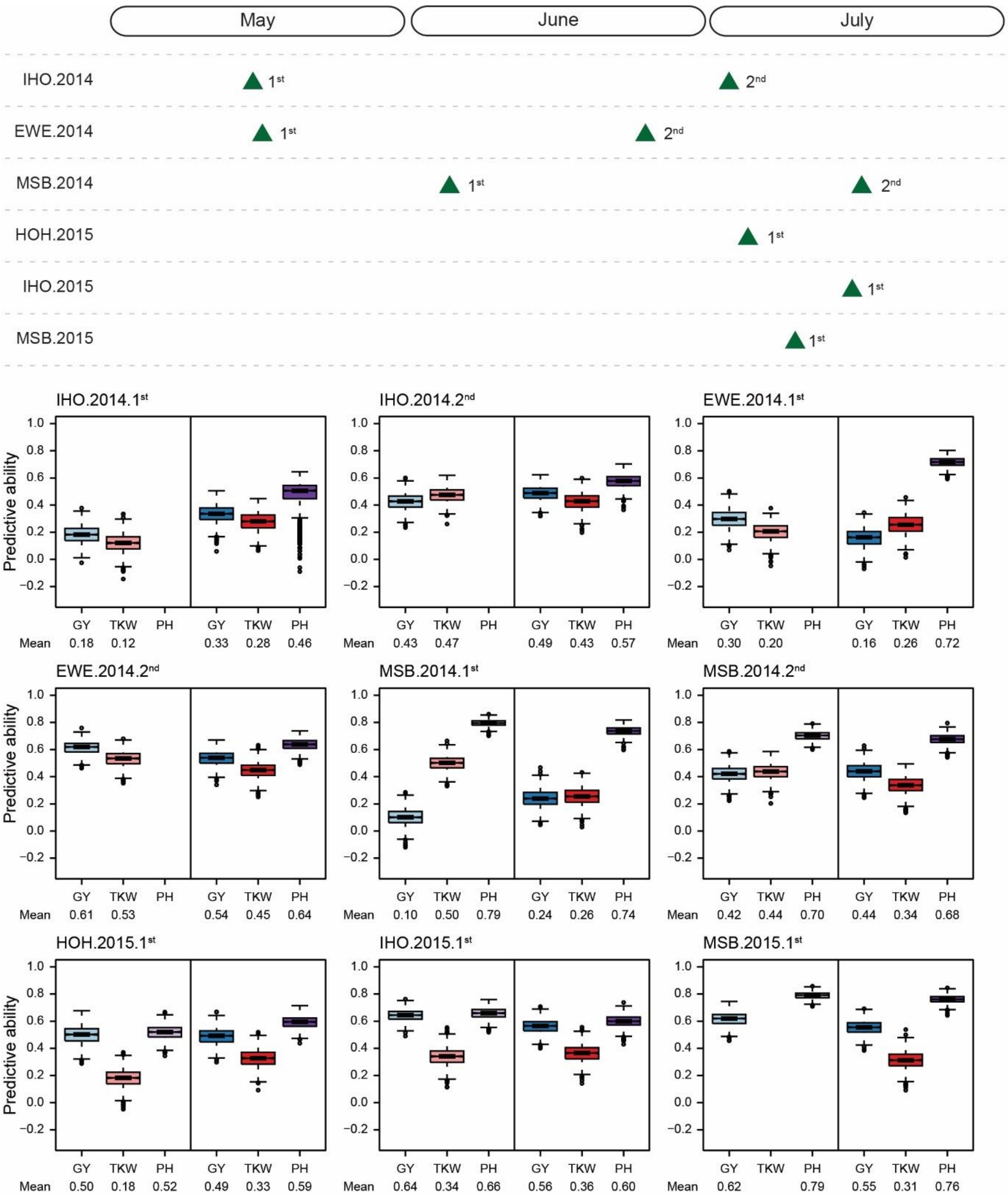

Fig. S4 Date of hyperspectral data collection (green triangles) in each environment, and boxplots of predictive ability of hyperspectral data for grain yield (GY), thousand-kernel weight (TKW) and plant height (PH) shown for phenotypic data BLUEs from the same environment as the hyperspectral data (left box) and BLUEs across all environments (right box).
